# Investigation of Combustion and NO/SO_2_ Emission Characteristics during the Co-Combustion Process of Torrefied Biomass and Lignite

**DOI:** 10.3390/molecules29122728

**Published:** 2024-06-07

**Authors:** Xu Yang, Wenkun Zhu, Zhaoming Li, Li Xu, Shujun Zhu, Jilin Tian, Zhuozhi Wang, Boxiong Shen

**Affiliations:** 1 School of Chemical Engineering, Hebei University of Technology, Tianjin 300401, China; 2 School of Energy Science and Engineering, Harbin Institute of Technology, Harbin 150001, China; 3 Anhui Special Equipment Inspection Institute, 45 Dalian Road, Hefei 230051, China; 4 Shanxi Key Laboratory of Coal Flexible Combustion and Thermal Conversion, Datong 037000, China

**Keywords:** co-combustion, combustion characteristic parameters, kinetic analysis, pollutant emission

## Abstract

This paper investigates the combustion characteristics and pollutant emission patterns of the mixed combustion of lignite (L) and torrefied pine wood (TPW) under different blending ratios. Isothermal combustion experiments were conducted in a fixed bed reaction system at 800 °C, and pollutant emission concentrations were measured using a flue gas analyzer. Using scanning electron microscopy (SEM) and BET (nitrogen adsorption) experiments, it was found that torrefied pine wood (TPW) has a larger specific surface area and a more developed pore structure, which can facilitate more complete combustion of the sample. The results of the non-isothermal thermogravimetric analysis show that with the TPW blending ratio increase, the entire combustion process advances, and the ignition temperature, maximum peak temperature, and burnout temperature all show a decreasing trend. The kinetic equations of the combustion reaction process of mixed gas were calculated by Flynn–Wall–Ozawa (FWO) and Kissinger–Akahira–Sunose (KAS) kinetic equations. The results show that the blending of TPW reduces the activation energy of the combustion reaction of the mixed fuel. When the TPW blending ratio is 80%, the activation energy values of the mixed fuel are the lowest at 111.32 kJ/mol and 104.87 kJ/mol. The abundant alkali metal ions and porous structure in TPW reduce the conversion rates of N and S elements in the fuel to NO and SO_2_, thus reducing the pollutant emissions from the mixed fuel.

## 1. Introduction

In recent years, the development of industries such as chemical engineering and electricity has led to a continuous increase in fossil fuel consumption, resulting in a rising demand for coal [1]. As a non-renewable resource, the total amount of coal is gradually decreasing. China’s reserves of brown coal, accounting for 40% of total coal reserves, are relatively abundant. However, brown coal itself contains rich active oxygen-containing functional groups, and its moisture content is very high, with a large proportion of ash content, leading to poor combustion performance [2]. Biomass energy is considered the fourth largest energy source, following coal, oil, and natural gas. China possesses abundant biomass energy resources, equivalent to approximately 460 million tons of standard coal. However, the current utilization capacity is less than 60 million tons, accounting for only 13% of the total reserves. Improper handling of biomass energy can pose significant threats to the environment [3]. The low energy density, low calorific value, high moisture content, and poor grindability of biomass limit its direct utilization as an energy source [4,5,6]. In order to enhance the characteristics of raw biomass materials, pre-treatment by torrefying biomass at 200–300 °C is conducted to remove volatile components and moisture, thereby increasing the fixed carbon content and calorific value. This process also improves its hydrophilicity and grindability to a certain extent [7,8]. Additionally, the more developed pore structure in torrefied biomass facilitates heat transfer and the transport of oxidants [9,10]. The research findings indicate that torrefied biomass combustion exhibits good thermal stability and high calorific value, reducing the risk of spontaneous combustion, and decreasing the emissions of pollutants such as SO_x_ and NO_x_. Therefore, direct combustion of biomass holds significant potential for the future [11,12]. The characteristics of biomass fuel after torrefying are very similar to those of lignite [13,14]. Blending biomass with lignite can improve the combustion performance of lignite and stabilize the combustion process [15,16].

Some scholars [17,18,19,20,21,22] have conducted experiments on the co-combustion of biomass and coal. The results show that blending biomass below 20% helps to enhance combustion reactivity, and the emission of pollutants (SO_x_ and NO_x_) decreases after co-combustion. The main reason is that the blending of biomass reduces the ignition temperature of coal, advancing the combustion process. A large number of alkali metal ions catalyze the combustion process, and biomass volatiles containing a large number of free radicals (CH_i_, NH_i_, H) promote the reduction reaction of NO_x_. Zhang et al. [23] conducted co-combustion experiments using pretreated miscanthus and lignite. The study found that blending miscanthus reduced the ignition temperature and burnout temperature during combustion, as well as the emission of pollutants while increasing the combustion rate. Guo et al. [18] conducted co-combustion experiments using biomass pellets with bituminous coal and lignite. The study found that the combustion process consisted of three stages: combustion of volatile matter in biomass, combustion of biochar, and combustion of coal. The blending of biomass primarily improved the third stage of combustion, and with an increasing blending ratio, the maximum combustion rate and combustion index increased, indicating that the addition of biomass improved the combustion performance of coal. Xiao et al. [24] conducted co-combustion experiments using hemicellulose, cellulose, and lignin with lignite separately. The research results indicate that free radicals in the volatile fractions of hemicellulose and cellulose react with the free radicals in lignite, promoting the decomposition of lignite. However, there are still certain knowledge gaps regarding the co-combustion characteristics of torrefied biomass and lignite. Therefore, it is imperative to fully understand the interactions between biomass char and coal-based fuels to enhance the combustion performance of mixed fuels and reduce pollutant emissions.

The purpose of this study is to investigate the combustion characteristics of torrefied pine wood (TPW) and lignite (L) during co-combustion, as well as the release characteristics of NO_x_ and SO_x_, to gain deeper insights into the interaction between torrefied biomass and lignite during co-combustion. The findings of this research can provide theoretical data for the separate combustion of biochar as well as its co-combustion with lignite, which is of significant importance for the clean and efficient utilization of agricultural solid waste and coal.

## 2. Experimental

### 2.1. Sample Preparation

The biomass material selected for this study was pine wood. The preparation process of torrefied pine wood (TPW) involved heating at 280 °C under a simulated flue gas atmosphere (6% O_2_ + 10% CO_2_ + 10% H_2_O + 74% Ar) for 30 min. The coal sample used was lignite (L) sourced from Inner Mongolia Autonomous Region. Prior to experimentation, both TPW and L samples were dried at 60 °C for 24 h to ensure thorough dehydration. Subsequently, they underwent a series of pretreatments, including grinding and sieving to prepare samples with particle sizes ranging between 90 and 125 μm. TPW and L were mixed in mass ratios of 0/1, 2/8, 4/6, 6/4, 8/2, and 1/0 to form blended samples, denoted as 0% TPW, 20% TPW, 40% TPW, 60% TPW, 80% TPW, and 100% TPW, respectively.

### 2.2. TG Analysis

Using a thermogravimetric analyzer (SDT Q600, TA Instruments, New Castle, DE, USA), the thermal decomposition behaviors of TPW and L were investigated. A sample of 10 mg was placed in an alumina crucible for mass loss testing. Under atmospheric air conditions, the sample was heated at a rate of 10 °C/min within a temperature range of 30 to 800 °C, with a gas flow rate of 100 mL/min. According to the national standard (GB/T 33304–2016), the ignition and burnout temperatures (*T*_i_ and *T*_b_) were determined using the tangent method [25]. *T*_m_ was the temperature corresponding to the maximum mass loss rate, and the comprehensive combustion index (*S*) was calculated as follows:

(1) $$S=\frac{DTG_{\max}\times DTG_{\mathrm{mean}}}{T_{b}\times T_{i}^{2}}$$

where *DTG*_max_ denotes the maximum mass loss rate of the sample; and *DTG*_mean_ denotes the mean mass loss rate of the sample.

### 2.3. Fixed-Bed Combustion Experiment System and Conditions

**Figure 1:**
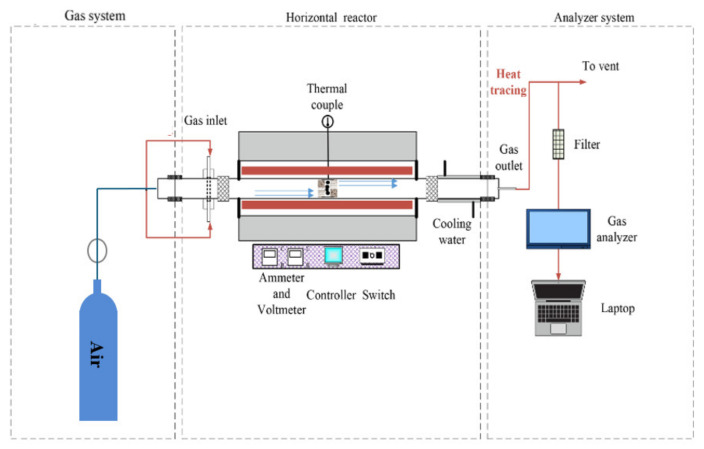
Fixed bed reaction system.

To investigate the emission characteristics of pollutants, experiments were conducted on the co-combustion of TPW and L using a fixed bed reaction system, as shown in Figure 1. The fixed bed reaction system consists of a gas supply system, a horizontal reaction system, and a gas analysis system. High-purity air was used as the experimental gas, controlled by a mass flow controller to maintain a constant flow rate of 1.5 L/min. In each experiment, approximately 0.3 ± 0.005 g of sample was first placed into a quartz boat and placed in the low-temperature zone outside the furnace. The reactor was then heated at a rate of 20 °C/min to the specified reaction temperature, with the experimental gas sweeping for 300 s. Subsequently, the quartz boat containing the sample was quickly transferred into the constant temperature reaction zone. The concentrations of NO and SO_2_ in the flue gas were monitored in real time using a flue gas analyzer (MCA 14 m, Hebi, China), with data recorded at a frequency of 10 s. The combustion reaction was considered finished when CO_2_ emissions undetected by the flue gas analyzer were observed.

After the combustion reactions of L and TPW, the total amount of NO and SO_2_ in the flue gas was calculated to determine the conversion rate of fuel nitrogen to NO and SO_2_. Integrating the NO and SO_2_ emission curves during the combustion process yielded the total generation of NO and SO_2_ in the flue gas. By combining this result with Equations (2) and (3), the conversion rate of NO and SO_2_ during the combustion process of the samples under different reaction conditions is determined.

(2) $$\eta_{NO}=\frac{1000M_{N}\times \frac{Q}{60\;\times \;22.4}\times \int_{0}^{t}(C_{NO}\times 10^{-6})dt}{m\times f_{N}}$$

(3) $$\eta_{SO_{2}}=\frac{1000M_{S}\times \frac{Q}{60\;\times \;22.4}\times \int_{0}^{t}(C_{i}\times 10^{-6})dt}{m\times f_{S}}$$

where *η*_NO_ and *η*_SO2_ represent the values of the specific conversion ratios of the nitrogen and sulfur-containing gaseous products, respectively (%); *M*_N_ and *M*_S_ are the molar mass values of the nitrogen element and sulfur element, respectively (g/mol); *Q* is the volumetric flow rate of the reaction gas (L/ min); *C*_NO_ represents the volumetric fraction of NO in the downstream gas (ppm); *C*_SO2_ represents the volumetric fraction of SO_2_ in the downstream gas (ppm); *m* is the mass of the sample (mg); *f*_N_ and *f*_S_ are the percentage of nitrogen/sulfur content in the sample, respectively.

### 2.4. Analy of Physical and Chemical Properties

The proximate analysis of the samples was conducted in accordance with the Chinese National Standards GB/T 28731-2012 and GB/T 212-2008 [26]. The ash and volatile content of the samples were measured using a muffle furnace, while the C, H, N, and S contents in the samples were analyzed using an elemental analyzer (vario MACRO cube, Anhalt, Germany). The high heating value (HHV) of the samples was determined using an oxygen bomb calorimeter (SDC712 XRY-1A). The surface morphology of the samples was observed using a scanning electron microscope (SEM). The specific surface area and pore structure of the samples were measured using BET (nitrogen adsorption).

### 2.5. Kinetics Analysis

The chemical kinetics occurring during the combustion reaction process are referred to as combustion reaction kinetics. Reaction kinetics is employed to assess chemical reaction rates and their influencing factors and to rationalize experimental results with a reaction mechanism. As the combustion reaction progresses, the combustion conversion rate of char can be described by two functions. One is the temperature function, denoted as *k*(*T*), and the other is the conversion function, denoted as *f*(*X*). Therefore, the conversion rate of the combustion reaction can be calculated using Equation (4):

(4) $$\frac{dX}{\mathrm{dt}}=k(T)f(X)$$

where t and *T* represent time and temperature, respectively. The conversion (*X*) of the sample during the combustion process can be calculated by the following formula:

(5) $$X=\frac{m_{0}-m_{t}}{m_{0}-m_{\infty }}$$

where *m*_0_ represents the initial mass of the sample, *m*_t_ represents the mass of the sample at time t, and *m*_∞_ represents the final mass of the sample.

The *k*(*T*) function, which reflects the relationship between temperature and reaction rate, is described by the Arrhenius equation.

(6) $$k(T)=Ae^{-E/RT}$$

where *A* is the pre-exponential factor, *E* is the activation energy, and R is the general gas constant (8.314 J/(mol/K)).

Since the temperature (*T*)can be determined by the heating rate (*β*) and time (t) of the combustion reaction, Equation (4) can be rewritten as follows:

(7) $$\frac{dX}{dT}=\frac{A}{\beta }\cdot \exp(-\frac{E}{RT})\cdot f(X)$$

The function *g*(*X*) as the integral form of *f*(*X*) can be expressed, and Equation (7) can be written as follows:

(8) $$g(X)=\frac{AE_{a}}{\beta R}\cdot h(u)$$

where *h*(*u*) is the approximate solution, and u is defined as follows:

(9) $$u=\frac{E}{R}$$

The combustion processes of biomass and coal are highly complex and cannot be represented by a single mechanism. In cases where the reaction mechanism functions are uncertain, the iso-conversion method can accurately calculate the activation energy of combustion reactions. The reaction rate in this calculation method depends solely on the reaction temperature. Therefore, the kinetic parameters of the combustion reaction are calculated using the Kissinger–Akahira–Sunose (KAS) and Flynn–Wall–Ozawa (FWO) models [26]. The calculation formula is as follows:

(10) $$\mathrm{KAS}:\ln\left(\frac{\beta }{T^{2}}\right)=\ln\left[\frac{AE}{Rg(X)}\right]-\frac{E}{RT}$$

(11) $$\mathrm{FWO}:\ln\left(\beta \right)=\ln\left[\frac{AE}{Rg(X)}\right]-5.331-\frac{E}{RT}$$

The calculation of the two activation energies can be performed using linear plot methods. In this study, 10 mg samples were chosen and subjected to TGA at different heating rates of 10, 15, and 20 K/min to determine the kinetic parameters of each sample.

## 3. Results and Discussion

### 3.1. Physicochemical Characteristics of Samples

In order to evaluate the combustion performance, Table 1 presents the ultimate analysis, proximate analysis, and HHV of TPW and L. The C content and FC content in L are 80.76% and 45.22%, respectively, both of which are higher than those in TPW. The volatile content of L is similar to TPW, but the higher content of H and O elements in TPW indicates that this sample is more prone to combustion [27]. Due to the lower C content in TPW, its HHV is 23.16 MJ/kg, but the HHV value of L is higher at 31.01 MJ/kg. The lower HHV suggests that less heat is released during the combustion process of the sample, which is also a drawback of replacing traditional coal-fired power generation with bio-fuel power generation.

**Figure 2:**
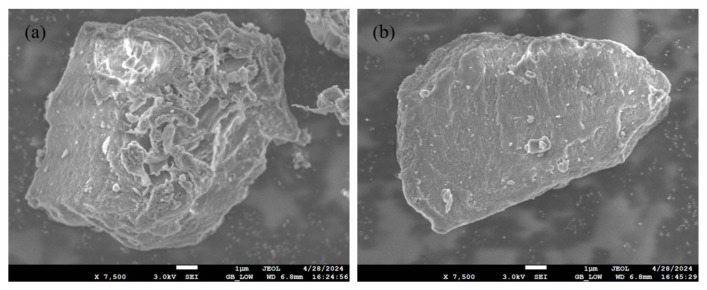
The SEM image results of (**a**) TPW-(**b**) L.

The physical properties of the samples also affect their combustion performance [26]. The results of SEM analysis of the microstructure of the fuel are shown in Figure 2. The surface of the TPW sample exhibits wrinkles, roughness, and obvious pores, which are caused by the loss of volatiles after torrefaction treatment, a result confirmed by Zhao et al. [5]. L particles have a block-like structure with a smooth surface and no apparent pore structure, showing some differences compared to TPW. To further illustrate the pore structure of each fuel, nitrogen adsorption tests were conducted to characterize the specific surface area, pore volume, and pore size distribution of the fuel, as shown in Table 2. The results show that the specific surface area of TPW is 16.462 m^2^/g, much higher than that of L at 7.265 m^2^/g. The pore volume and pore size distribution of TPW are also higher than those of L. Generally, the specific surface area and pore volume are the main factors affecting the combustion performance of samples. A larger specific surface area increases the possibility of interaction with oxygen and facilitates the transfer of heat during the combustion process, thereby improving the combustion reaction rate [28].

### 3.2. The Thermochemical Results of Individual Fuel

**Figure 3:**
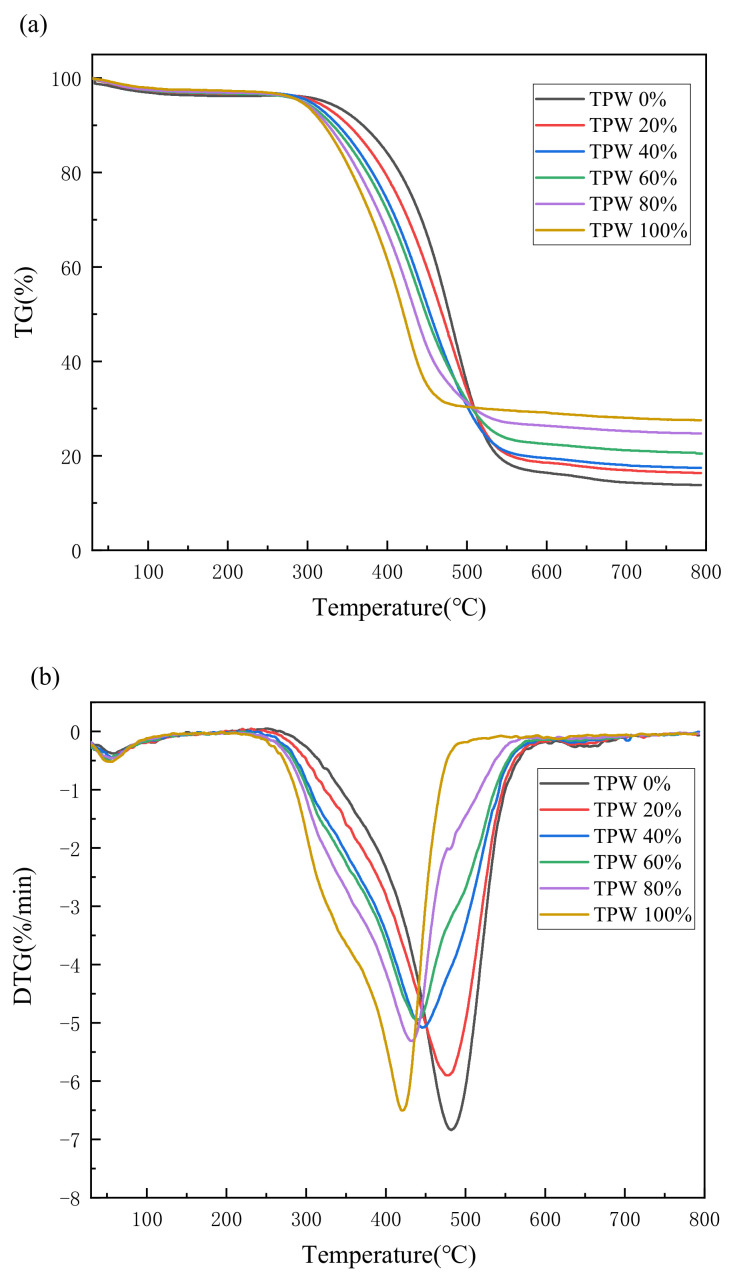
TG (**a**)-DTG (**b**) curves of different samples.

Thermogravimetric analysis is an effective method for studying the combustion characteristics of fuels. Analysis of TG and DTG curves can determine the combustion characteristic parameters of each solid sample [29]. The TG curves of TPW, L, and their blend combustion at a heating rate of 10 °C/min are illustrated in Figure 3. As the combustion temperature increases, the thermal decomposition of both fuels leads to an increase in weight loss. The combustion process mainly consists of two stages: dehydration and char combustion. This is different from raw pine wood, which typically undergoes a volatile combustion stage. Due to low-temperature oxygen-deficient pretreatment, the volatile fraction in biochar releases with lower activity, resulting in the overlap of volatile fraction and char combustion stages. Additionally, the ash content in TPW is higher than that in L, leading to more residue after combustion. Generally, the evolution range of the first stage extends from 30 °C to 150 °C, attributed to the evaporation of moisture in the fuel, while the second stage spans from 250 °C to 550 °C, representing the combustion of volatile matter and fixed carbon.

TPW and L exhibit differences in combustion behavior. The first stage involves dehydration, where all samples show very consistent weight change curves. However, significant differences emerge in the second stage. The combustion range of TPW mainly occurs between 250 and 500 °C, while L exhibits a delayed combustion phenomenon with a range between 270 and 550 °C. TPW primarily consists of hemicellulose, cellulose, and lignin, where lignin is relatively stable, and hemicellulose and cellulose mainly consist of polysaccharides with low polymerization degrees, abundant oxygen-containing active functional groups, and higher O/C atomic ratios (approximately 0.82 for hemicellulose and 0.83 for cellulose). This results in strong combustion reactivity, making them easy to ignite and burn out [27]. The organic components in L are relatively stable, mainly composed of thermally stable polycyclic aromatic structures and long-chain macromolecules, with higher thermal decomposition temperatures.

During the stages of volatile release and char combustion, TPW and L exhibit distinct combustion behaviors. The maximum peak of TPW is slightly lower than that of L, but the corresponding temperature, *T*_m_ is lower in TPW. This is primarily because this peak mainly results from the combustion of volatile and fixed carbon. While the volatile content in TPW is similar to L, the fixed carbon content is slightly lower in TPW. Hence, the peak is slightly lower in TPW compared to L. However, TPW contains abundant oxygen-containing active functional groups, and the catalytic effect of alkali metal ions such as Na and K promotes the combustion reaction, leading to the lower *T*_m_.

### 3.3. Combustion Characteristics of Fuel Blends

**Figure 4:**
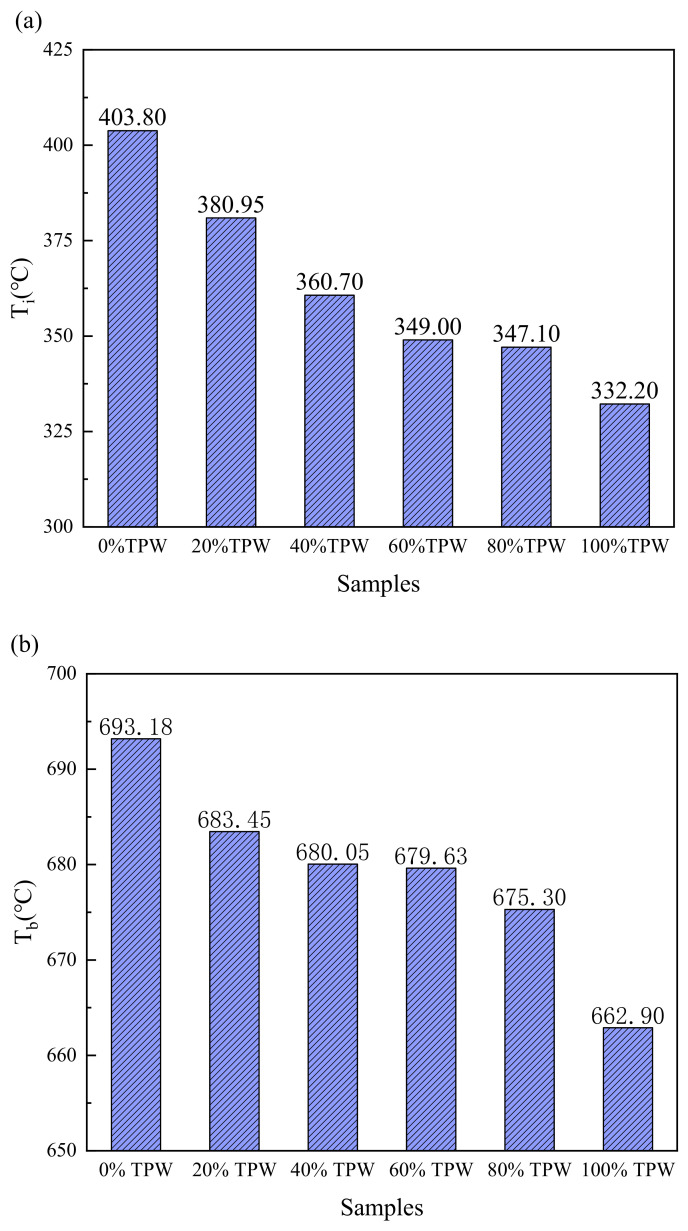

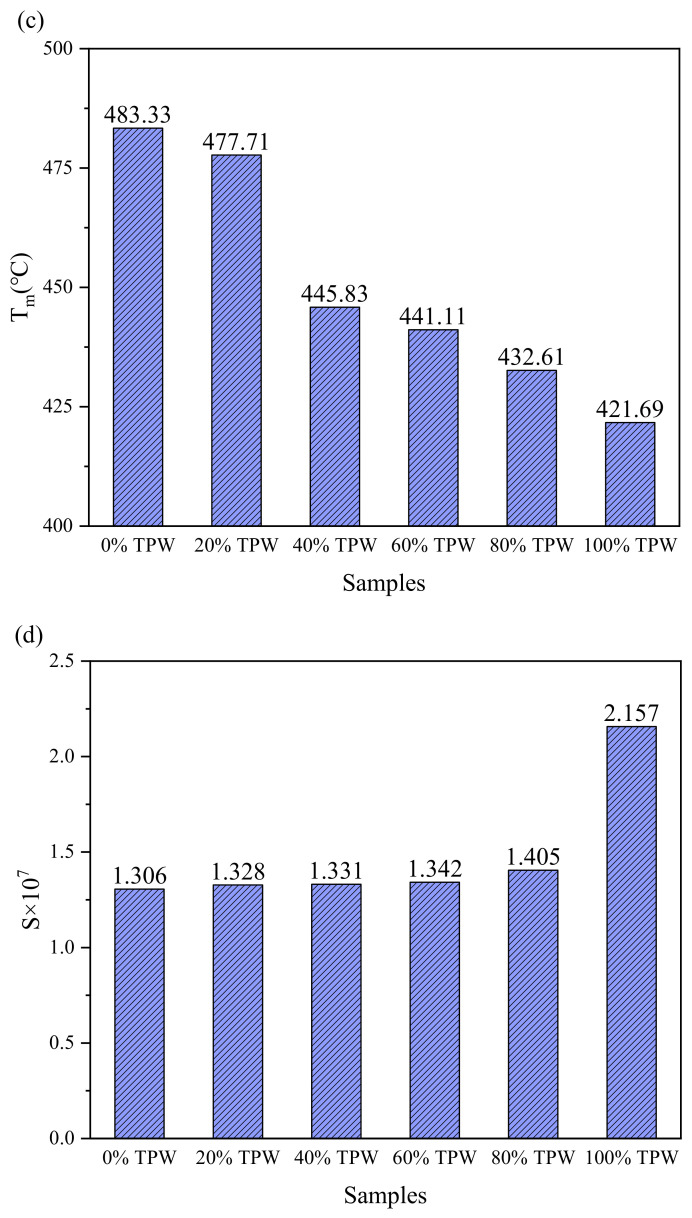
Combustion characteristic parameters for each sample; (**a**–**d**) the ignition temperature, burnout temperature, maximum weight loss temperature, and comprehensive combustion index of each sample.

To investigate the role of TPW in the co-combustion process, the combustion processes of different proportion mixtures of fuels were studied. Figure 3 shows the combustion reaction curve of the mixed samples at a heating rate of 10 °C/min. Figure 4 presents the characteristic parameters of the combustion reactions of the six samples. The combustion curve of the mixture falls between the combustion curves of TPW and L. As the proportion of TPW in the mixture increases, the combustion curve of the mixed gas gradually shifts toward the low-temperature zone. Due to the slightly higher ash content of TPW than L, the residual substances after combustion gradually increase, indicating a close correlation between the combustion of the mixed gas and that of individual samples [30].

All combustion curves exhibit double peaks, with the range of 30–100 °C representing the dehydration stage and 250–550 °C representing the combustion stages of volatile matter and fixed carbon. As the mixing ratio of L increases, the DTG peak shifts toward the high-temperature region. From Figure 4c, it can also be observed that the *T*_m_ of TPW combustion is 421.69 °C, while that of L combustion is higher at 483.33 °C. The *T*_m_ of the combustion for mixed samples falls between the two, indicating that the combustion of mixed samples is influenced by both TPW and L. With the decrease in the biomass mixing ratio, the peak value of the second stage of combustion shows a trend of initially decreasing and then increasing. This is mainly due to the different temperature zones where the main components of TPW and L undergo chemical reactions, resulting in a broader peak width and a corresponding decrease in peak value for the combustion of mixed fuels. The blending of TPW accelerates the combustion of volatile and fixed carbon in L, promoting an early combustion process, which is consistent with previous research results [31].

Figure 4 reveals that with the increase in TPW mixing ratio, the ignition temperature and burnout temperature of the mixed fuel decrease sequentially, and the temperature corresponding to the maximum combustion rate also shows a corresponding trend. Compared to pure L, when the TPW mixing ratio is 0.8, *T*i decreases by 56.70, *T*_b_ decreases by 17.88, and *T*_m_ decreases by 250.72. The decrease in *T*_i_ is mainly due to the lower *T*_i_ of the fiber components in TPW, which start burning and release heat at lower temperatures, raising the temperature of the mixture and promoting the release and combustion of volatile matter in L, thereby advancing the combustion process. Thus, the content of TPW plays a dominant role in promoting the combustion process. However, lignite mainly consists of large molecules and aromatic structures, possesses strong thermal stability, and contains only a small amount of alkali and alkaline earth metals (AAEMs) compared to TPW, resulting in poorer combustion reactivity compared to TPW and leading to higher ignition and burnout temperatures. This indicates that the addition of TPW can promote the combustion performance of L and also illustrates the complexity of co-combustion reactions between TPW and L. However, the changes in *T*_i_, *T*_b_, and *T*_m_ are not linear, suggesting the possible presence of synergistic effects in the combustion reaction process between TPW and L [26]. This is consistent with the research results of Wang and Xiao et al. [19,24].

To evaluate the entire combustion process, we calculated the comprehensive combustion index. The results show that the comprehensive combustion index of both L and mixed fuels is very close, ranging from 1.306 to 1.405. However, the *S* value for TPW combustion alone is significantly higher, reaching 2.157. This is mainly due to the addition of L, which increases the ignition temperature and burnout temperature. The trend of the comprehensive combustion index is consistent with that of *T*_i_, TPW, and *T*_m_. As the TPW mixing ratio increases, the comprehensive combustion index gradually decreases, which is consistent with previous research results [18,32].

### 3.4. Synergistic Effects of Co-Combustion Reaction

In this study, the blending of TPW significantly improved the combustion performance of L, promoting earlier ignition and burnout, indicating a clear interaction between the two fuels. To verify the interaction between TPW and L during the combustion process, a synergistic factor (*SF*) was quantified using the peak temperature and burnout temperature in the reaction zone, as well as the required time. When *SF* > 1.15, the mixture exhibited a significant synergistic effect; when 0.8 < *SF* < 1.15, the synergistic effect was not significant, and when *SF* < 0.8, it indicated a deterioration in the combustion performance of the mixture. The formula for the synergistic effect parameter is as follows [33]:

(12) $$SF=\frac{SI_{blend}}{SI_{coal}}$$

The synergistic index (*SI*) can be calculated using the following formula:

(13) $$SI=\frac{1}{t_{p-s}^{0.5}T_{b}^{2}T_{m}}\times 10^{6}$$

**Figure 5:**
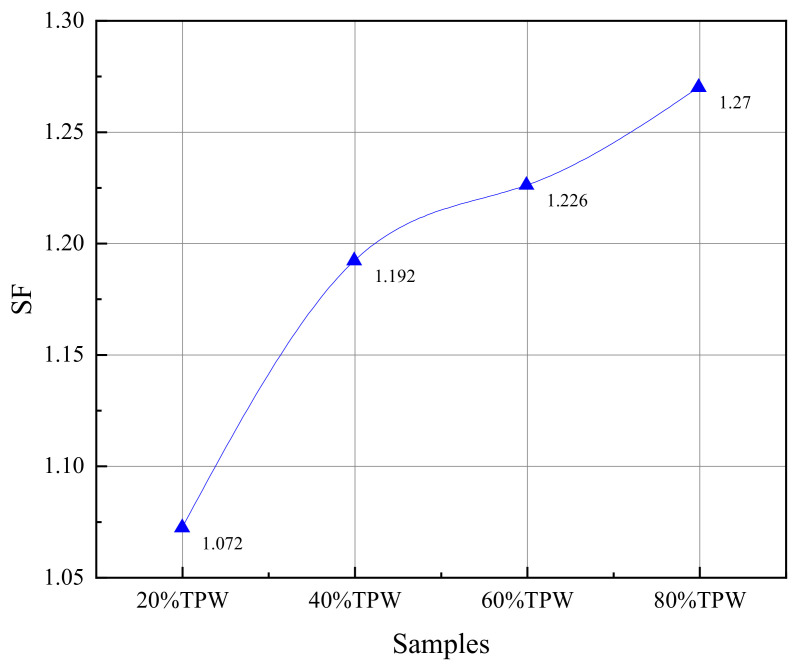
The *SF* of samples with different mixing ratios.

The values of *SF* under various mixing ratios are shown in Figure 5. It can be observed that the synergistic coefficient increases with the increase in the blending ratio. When the biomass blending ratio is 0.2, the minimum *SF* is 1.07, indicating less apparent interaction. This is mainly because the relatively small proportion of TPW lacks sufficient alkali metal elements—such as K, Ca, and Mg—to catalyze the combustion process. However, as the blending ratio increases, better interactions are demonstrated. When the biomass blending ratio is 0.4, the *SF* increases to 1.19, reaching 1.22 at a blending ratio of 0.6. The catalytic effect is most pronounced at the maximum TPW blending ratio, exhibiting the best synergistic effect with *SF* reaching 1.27. Of course, the reason for the synergistic effect is not only due to the catalysis of oxidation reactions by abundant AAEMs in biochar. The main components of TPW are highly active semi-cellulose and cellulose, which readily undergo exothermic oxidation reactions, compensating for the energy required for the oxidative combustion of L during the combustion process, thus achieving the early combustion of L. Moreover, TPW has a very low moisture content and abundant pore structure, which accelerates the combination of oxidants with active sites on the particle surface. This reduces the diffusion resistance required for oxidative molecule adsorption and desorption, thereby improving the combustion performance of the mixed fuel [18,34].

### 3.5. Kinetics Analysis

**Figure 6:**
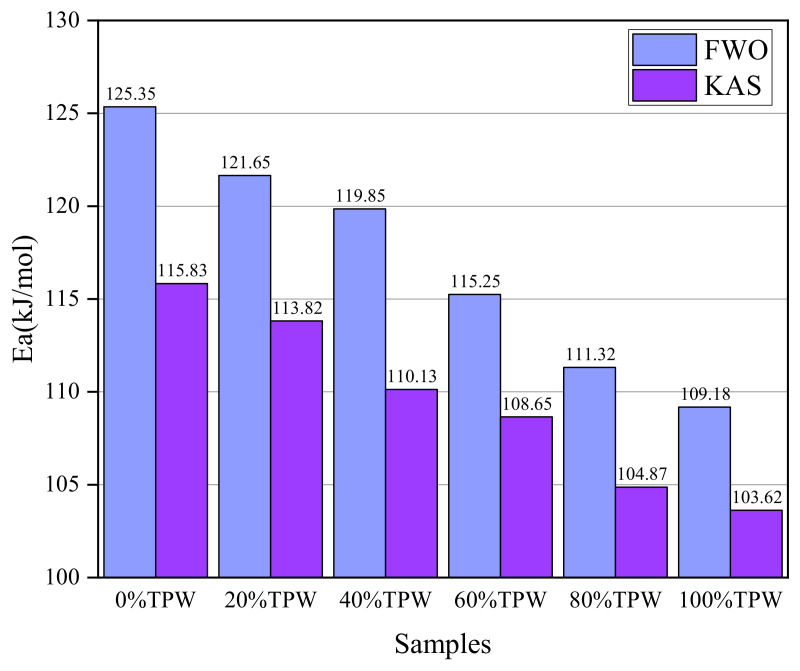
Calculation of activation energies for different samples.

In order to analyze the kinetic performance of TPW and L co-combustion, the combustion activation energies of six samples were calculated using the FWO and KAS methods at three different heating rates (10, 15, and 20 °C/min). The activation energy results are summarized in Figure 6. Both types of activation energy exhibit the same trend, where the activation energy is the highest for L combustion alone and the lowest for TPW combustion alone, decreasing sequentially with an increase in the TPW mixing ratio. Using the FWO method, the average combustion activation energies for the six samples are 125.35 kJ/mol, 121.65 kJ/mol, 119.85 kJ/mol, 115.25 kJ/mol, 111.32 kJ/mol, and 109.18 kJ/mol, respectively. In the calculation process using the KAS method, the average combustion activation energies for the six samples are 115.83 kJ/mol, 113.82 kJ/mol, 110.13 kJ/mol, 108.65 kJ/mol, 104.87 kJ/mol, and 103.62 kJ/mol, respectively. The calculated activation energy results indicate that blending TPW with L favors the combustion of L, mainly due to the smaller O/C and H/C ratios in L, which results in fewer C-H and C-O bonds and more C-C bonds. Breaking C-C bonds requires more energy than breaking C-H and C-O bonds. The lower activation energy of the samples indicates that they can reach the energy required for chemical reactions at lower temperatures, consistent with the results mentioned earlier, namely, the addition of TPW shifts the thermogravimetric curves of the mixed samples toward lower temperatures [34].

### 3.6. NO Emission Character

Combustion experiments were conducted using a fixed bed reaction system to evaluate the NO emissions during the combustion processes of TPW, L, and their mixtures. The calculation of the conversion rate of N to NO in the fuel indicates the interaction between TPW and L in co-combustion. Experimental results demonstrate that blending different proportions significantly impacts the NO emissions of the mixed fuel.

**Figure 7:**
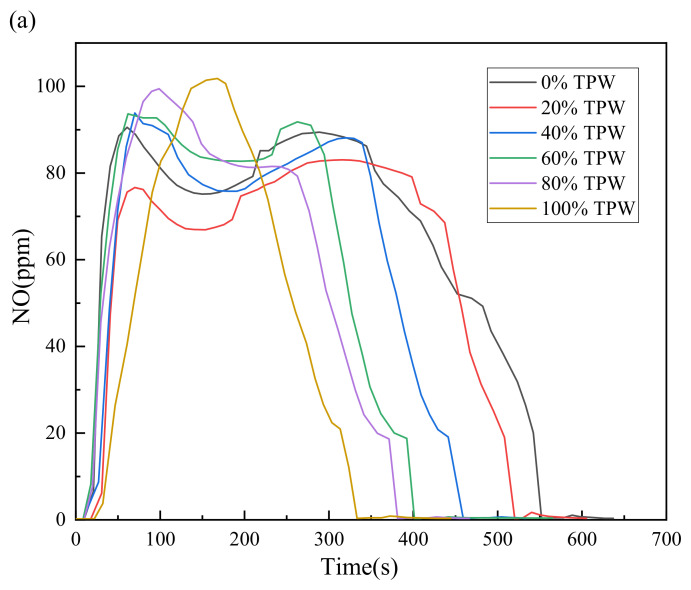

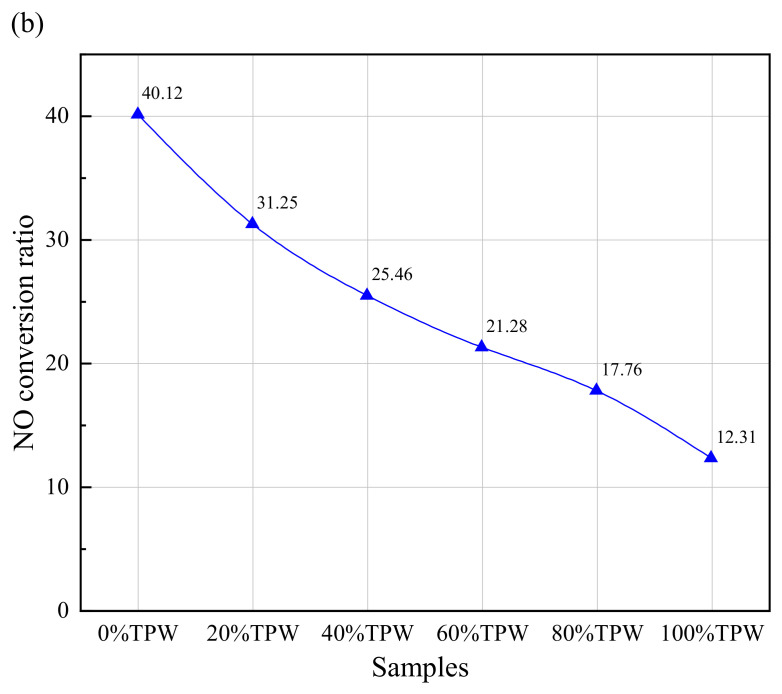
NO emission pattern during combustion, (**a**) emission curve, (**b**) NO conversion rate.

The concentration variation curve of NO in the mixed fuel over time is depicted in Figure 7a. Due to the higher reactivity and faster reaction rate of TPW, the NO emission peak exhibits a single-peak pattern with TPW having the largest peak. In contrast, the emission curves of NO for L and their blends exhibit a double-peak pattern. The first peak is attributed to the combustion of volatiles generating NO, and its magnitude is influenced by the blending ratio of TPW; higher blending ratios result in larger peaks. The second peak is generated by the combustion of char, and although its magnitude does not significantly change with a decreasing TPW blending ratio, the emission range notably widens. This indicates that the blending of L prolongs and broadens the emission of NO. However, these findings are consistent with previous research results [35]. When L combusts alone, the widest peak is observed, and as the L blending ratio decreases, the peak width gradually narrows. This indicates that the blending of TPW promotes the early release of NO. Furthermore, the significant oxygen consumption during the early stages of TPW combustion leads to a rapid transition of the combustion atmosphere to oxygen-deficient conditions, generating new reducing volatile NH_3_. This may undergo significant gas-gas reactions with L during co-combustion, thereby promoting the reduction of NO. On the other hand, the reducing atmosphere supports the formation of biochar, facilitating the adsorption and heterogeneous reduction of NO generated during L combustion.

To better illustrate the interaction between TPW and L co-combustion on NO emissions, the conversion rate of fuel N to NO during the combustion process was measured at different blending ratios, as summarized in Figure 7b. The results indicate that the conversion rate of N to NO during L combustion alone is the highest at 40.12%. As TPW blending increases, the conversion rate gradually decreases, ranging from 31.25% to 17.76% for mixed fuels, with the lowest conversion rate of 12.31% during TPW combustion alone. The conversion rate of fuel nitrogen to NO mainly depends on the rates of NO generation and decomposition, which are significantly correlated with the blending ratio. The main factor affecting NO release is the reduction reaction of NO. The strength of this reduction reaction directly affects the release of NO during the combustion process. The main nitrogen precursor in TPW is N-A, which research indicates generates a significant amount of reducing NH_3_ during the volatile release stage [36]. In addition to the abundant AAEMs in TPW, which catalyze the reduction of NH_3_ to NO, significantly reducing the conversion of fuel nitrogen to NO, this is the main reason for the decreased NO conversion rate after TPW blending. Secondly, the surface of TPW particles has abundant active sites that easily combine with O_2_ in the reaction atmosphere to generate many oxygen-containing precursors, which further decompose into CO, CO_2_, and new active sites with stronger reactivity as the reaction proceeds [37]. Among them, CO_2_ does not participate in the reduction of NO, while the reducible gaseous product CO and the new active sites both undergo reduction reactions with NO, releasing fuel nitrogen in the form of N_2_, thereby reducing the conversion rate of NO in the mixed fuel [38,39]. Therefore, increasing the blending ratio of TPW can reduce the conversion rate of fuel nitrogen to NO.

### 3.7. SO_2_ Emission Character

**Figure 8:**
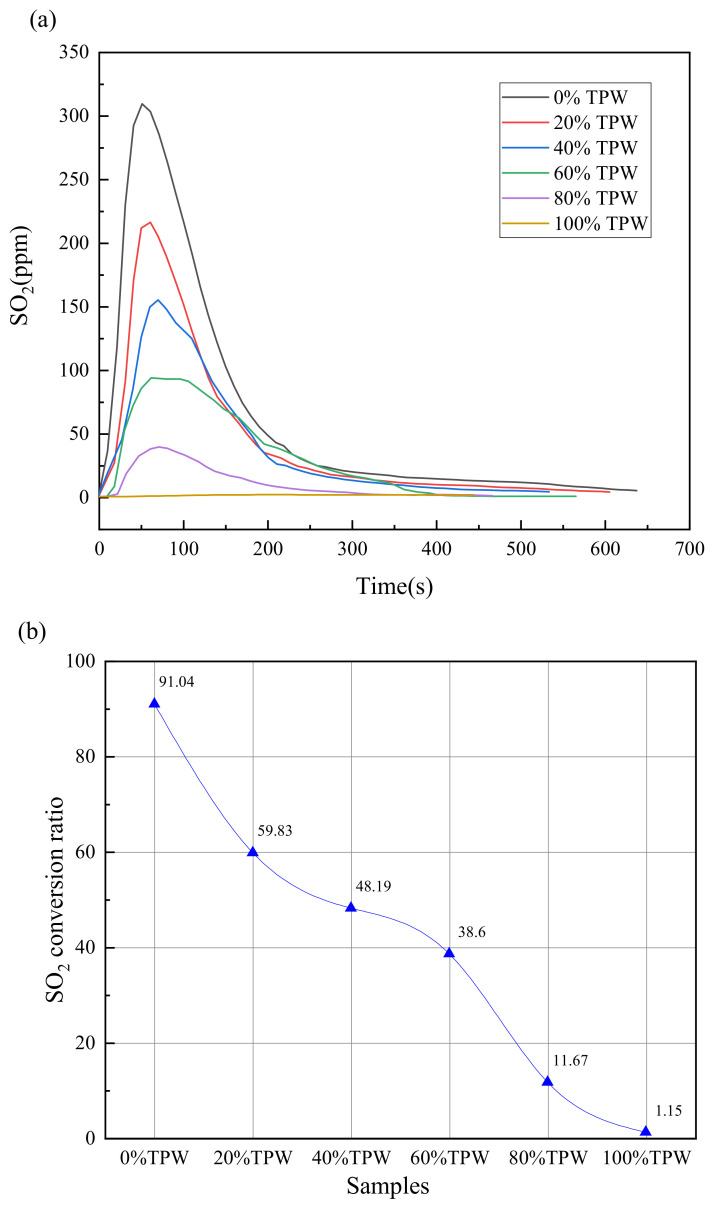
SO_2_ emission pattern during combustion, (**a**) emission curve, (**b**) SO_2_ conversion rate.

The influence of the blending ratio on the concentration and emission of SO_2_ in mixed fuels is illustrated in Figure 8a. Since the proportion of sulfur elements in TPW is very low, the emission of SO_2_ is almost zero when burned alone. For all other blending ratios of mixed fuels, the SO_2_ emission curve shows only one peak. As the blending ratio of TPW increases, the peak gradually decreases. The peak value for L is 309.2 ppm, while at a ratio of 80%, it decreases to 216.1 ppm, at a ratio of 60%, it decreases to 154.8 ppm, at a ratio of 40%, it decreases to 93.6 ppm, and at a ratio of 20%, the conversion rate is 39.2. The highest value for TPW is 1.6 ppm. The main reason for the decrease in value is the decrease in the proportion of sulfur elements in the mixed fuel as the blending ratio of TPW increases. All SO_2_ emission peaks occur at approximately 50 s and are high and narrow, which is attributed to the preheating decomposition of organic sulfur and some pyrite in the volatile fraction of the mixed fuel [40]. The release of SO_2_ is mainly concentrated in the first 200 s, and the emission curve from 200 s to the end of combustion is relatively flat, with concentrations all below 50 ppm. This is mainly related to the decomposition of sulfates in the fuel [41].

To analyze the interaction of blended combustion on SO_2_ emissions, the conversion rate of S to SO_2_ was calculated, and the results are shown in Figure 8b. The results indicate that L has the highest conversion rate, reaching 91.04%. When TPW is at a ratio of 0.2, the conversion rate is 59.83%, at a ratio of 0.4, it is 48.19%, at a ratio of 0.6, it is 38.6%, and at a ratio of 0.8, it is 11.67%. The conversion rate for TPW burning alone is 1.15%. It can be clearly seen that the blending of TPW significantly reduces the conversion rate of S to SO_2_, indicating a significant interaction between TPW and L, with the strength of the interaction gradually increasing with the blending ratio of biomass. This may be due to the rapid combustion of volatile components in TPW, which creates a localized oxygen-deficient atmosphere on the fuel surface, limiting the combustion rate of carbon. This allows the carbon to maintain a relatively intact pore structure in the early stages of combustion. AAEMs attached to the carbon skeleton can promote the absorption of SO_2_, fixing sulfur in the form of sulfates and sulfides. Therefore, when the blending ratio of TPW is relatively high, it exhibits good sulfur fixation effects [42].

## 4. Conclusions

This study investigates the co-combustion performances of TPW and L at different blending ratios. The results indicate that blending TPW enhances the combustion performance of the mixed fuel. Moreover, with an increase in the blending ratio of TPW, the combustion performance improves further, with higher comprehensive combustion indices and lower combustion activation energy. The results of the synergistic effect analysis show that the synergistic effect index is the highest at a blending ratio of 80%. This is primarily due to the enhanced porous structure facilitating the transfer of oxidizing molecules and heat and the abundant AAEMs in TPW promoting redox reactions during combustion. At the same time, the conversion rates of N and S pollutants released also decrease accordingly.

## Figures and Tables

**Table 1:** Ultimate analysis, proximate analysis, and high heating value results of the samples.

Samples	Ultimate Analysis (daf) (wt.%)					Proximate Analysis (ar)(wt.%)				HHV(MJ/kg)
	C	H	O ^a^	N	S	FC	Ash	Volatile	M_ar_	
TPW	61.33	4.92	32.76	0.98	0.01	33.21	30.86	33.27	2.66	23.16
L	80.67	3.95	14.17	0.56	0.65	45.22	18.9	33.37	2.51	31.01

^a^ Determined by difference; daf. Dry-ash-free basis; ar. As receive basis.

**Table 2:** Pore parameters of the samples.

Sample	Surface Area_BET_ (m^2^/g)	Pore Volume (cm^2^/g)	Pore Diameter_BJH_ (nm)
TPW	16.462	0.026	11.724
L	7.265	0.024	9.693

## Data Availability

The authors do not have permission to share data.

## Abbreviations

The following abbreviations are used in this manuscript:

L	lignite
TPW	torrefied pine wood
FWO	Flynn–Wall–Ozawa
KAS	Kissinger–Akahira–Sunose
*T*i	ignition temperature
*T*b	burnout temperature
*S*	comprehensive combustion index
HHV	high heating value
TGA	non-isothermal thermogravimetric analysis
AAEMs	alkali and alkaline earth metals

## References

[1] Chen W.-H., Lin B.-J., Lin Y.-Y., Chu Y.-S., Ubando A.T., Show P.L., Ong H.C., Chang J.-S., Ho S.-H., Culaba A.B. (2021). Progress in Biomass Torrefaction: Principles, Applications and Challenges. Prog. Energy Combust. Sci..

[2] Qi J., Fan C., Wu H., Li S. (2022). Structure Evolution of Lignite Char in Step Pyrolysis and Its Combustion Reactivity. Fuel.

[3] Yao J. (2023). Biomass energy: Only through diversified development can we go further. China Energy News.

[4] Parvej A.M., Rahman M.A., Reza K.M.A. (2022). The Combined Effect of Solar Assisted Torrefaction and Pyrolysis on the Production of Valuable Chemicals Obtained from Water Hyacinth Biomass. Clean. Waste Syst..

[5] Zhao Z., Feng S., Zhao Y., Wang Z., Ma J., Xu L., Yang J., Shen B. (2022). Investigation on the Fuel Quality and Hydrophobicity of Upgraded Rice Husk Derived from Various Inert and Oxidative Torrefaction Conditions. Renew. Energy.

[6] Zhang L., Wang Z., Ma J., Kong W., Yuan P., Sun R., Shen B. (2022). Analysis of Functionality Distribution and Microstructural Characteristics of Upgraded Rice Husk after Undergoing Non-Oxidative and Oxidative Torrefaction. Fuel.

[7] Wang Z., Zhang L., Xiong L., Xu L., Yang J., Shen B. (2023). Study on the regulation mechanism of torrefaction pretreat-ment on fuel quality and pyrolysis characteristics of rice husk. J. Fuel Chem. Technol..

[8] Kim H., Yu S., Ra H., Yoon S., Ryu C. (2023). Prediction of Pyrolysis Kinetics for Torrefied Biomass Based on Raw Biomass Properties and Torrefaction Severity. Energy.

[9] Ivanovski M., Goricanec D., Krope J., Urbancl D. (2022). Torrefaction Pretreatment of Lignocellulosic Biomass for Sustainable Solid Biofuel Production. Energy.

[10] Lu H., Gong Y., Areeprasert C., Ding L., Guo Q., Chen W.-H., Yu G. (2021). Integration of Biomass Torrefaction and Gasification Based on Biomass Classification: A Review. Energy Technol..

[11] Zhu X., Zhou S., Zhang Z., Zhang Y., Li J., Ahmed S., Yan B., Chen G., Li N. (2021). Flue Gas Torrefaction of Distilled Spirit Lees and the Effects on the Combustion and Nitrogen Oxide Emission. Bioresour. Technol..

[12] Munir S., Nimmo W., Gibbs B.M. (2011). The Effect of Air Staged, Co-Combustion of Pulverised Coal and Biomass Blends on NOx Emissions and Combustion Efficiency. Fuel.

[13] Liu Y., Rokni E., Yang R., Ren X., Sun R., Levendis Y.A. (2021). Torrefaction of Corn Straw in Oxygen and Carbon Dioxide Containing Gases: Mass/Energy Yields and Evolution of Gaseous Species. Fuel.

[14] Chen W.-H., Peng J., Bi X.T. (2015). A State-of-the-Art Review of Biomass Torrefaction, Densification and Applications. Renew. Sustain. Energy Rev..

[15] Gao M., Cheng C., Miao Z., Wan K., He Q. (2023). Physicochemical Properties, Combustion Kinetics and Thermodynamics of Oxidized Lignite. Energy.

[16] Gürel B., Kurtuluş K., Yurdakul S., Karaca Dolgun G., Akman R., Önür M.E., Varol M., Keçebaş A., Gürbüz H. (2024). Combustion of Chicken Manure and Turkish Lignite Mixtures in a Circulating Fluidized Bed. Renew. Sustain. Energy Rev..

[17] Cao J., Zhang R., Shi B., Shi M., Zhang L., Liu D. (2023). The Study of Co-Combustion Characteristics of Coal and Duckweed by Single Particle and TGA Methods. Powder Technol..

[18] Guo F., He Y., Hassanpour A., Gardy J., Zhong Z. (2020). Thermogravimetric Analysis on the Co-Combustion of Biomass Pellets with Lignite and Bituminous Coal. Energy.

[19] Wang S., Zou C., Lou C., Yang H., Pu Y., Luo J., Peng C., Wang C., Li Z. (2022). Influence of the Synergistic Effects between Coal and Hemicellulose/Cellulose/Lignin on the Co-Combustion of Coal and Lignocellulosic Biomass. Fuel.

[20] Yu D., Chen M., Wei Y., Niu S., Xue F. (2016). An Assessment on Co-Combustion Characteristics of Chinese Lignite and Eucalyptus Bark with TG–MS Technique. Powder Technol..

[21] Xinjie L., Singh S., Yang H., Wu C., Zhang S. (2021). A Thermogravimetric Assessment of the Tri-Combustion Process for Coal, Biomass and Polyethylene. Fuel.

[22] Yi B., Chen M., Gao Y., Cao C., Wei Q., Zhang Z., Li L. (2023). Investigation on the Co-Combustion Characteristics of Multiple Biomass and Coal under O_2_/CO_2_ Condition and the Interaction between Different Biomass. J. Environ. Manag..

[23] Zhang Y., Zahid I., Danial A., Minaret J., Cao Y., Dutta A. (2021). Hydrothermal Carbonization of Miscanthus: Processing, Properties, and Synergistic Co-Combustion with Lignite. Energy.

[24] Xiao Z., Wang S., Luo M., Cai J. (2022). Combustion Characteristics and Synergistic Effects during Co-Combustion of Lignite and Lignocellulosic Components under Oxy-Fuel Condition. Fuel.

[25] Lu J.-J., Chen W.-H. (2015). Investigation on the Ignition and Burnout Temperatures of Bamboo and Sugarcane Bagasse by Thermogravimetric Analysis. Appl. Energy.

[26] Liang W., Jiang C., Wang G., Ning X., Zhang J., Guo X., Xu R., Wang P., Ye L., Li J. (2022). Research on the Co-Combustion Characteristics and Kinetics of Agricultural Waste Hydrochar and Anthracite. Renew. Energy.

[27] Mo W., Wu Z., He X., Qiang W., Wei B., Wei X., Wu Y., Fan X., Ma F. (2021). Functional Group Characteristics and Pyrolysis/Combustion Performance of Fly Ashes from Karamay Oily Sludge Based on FT-IR and TG-DTG Analyses. Fuel.

[28] Fatehi H., Bai X.-S. (2017). Structural Evolution of Biomass Char and Its Effect on the Gasification Rate. Appl. Energy.

[29] Cahyanti M.N., Doddapaneni T.R.K.C., Kikas T. (2020). Biomass Torrefaction: An Overview on Process Parameters, Economic and Environmental Aspects and Recent Advancements. Bioresour. Technol..

[30] Mundike J., Collard F.-X., Görgens J.F. (2018). Co-Combustion Characteristics of Coal with Invasive Alien Plant Chars Prepared by Torrefaction or Slow Pyrolysis. Fuel.

[31] Wang P., Wang G., Zhang J., Lee J.-Y., Li Y., Wang C. (2018). Co-Combustion Characteristics and Kinetic Study of Anthracite Coal and Palm Kernel Shell Char. Appl. Therm. Eng..

[32] Jia W., Guo Y., Guo F., Li H., Li Y., Zhang Y., Wu J., Si C. (2023). Co-Combustion of Carbon-Rich Fraction from Coal Gasification Fine Slag and Biochar: Gas Emission, Ash Sintering, Heavy Metals Evolutions and Environmental Risk Evaluation. Chem. Eng. J..

[33] Oladejo J.M., Adegbite S., Pang C.H., Liu H., Parvez A.M., Wu T. (2017). A Novel Index for the Study of Synergistic Effects during the Co-Processing of Coal and Biomass. Appl. Energy.

[34] Zhang J., Zhang K., Huang J., Feng Y., Yellezuome D., Zhao R., Chen T., Wu J. (2024). Synergistic Effect and Volatile Emission Characteristics during Co-Combustion of Biomass and Low-Rank Coal. Energy.

[35] Wang Y., Jia L., Guo B., Shen X., Zheng X., Xiang J., Jin Y. (2022). Investigation of Interaction Mechanisms during Co-Combustion of Sewage Sludge and Coal Slime: Combustion Characteristics and NO/SO2 Emission Behavior. Sci. Total Environ..

[36] Wang Y., Jia L., Guo B., Wang B., Zhang L., Zheng X., Xiang J., Jin Y. (2022). N Migration and Transformation during the Co-Combustion of Sewage Sludge and Coal Slime. Waste Manag..

[37] Liu Y., Tan W., Liang S., Pan X. (2024). Study on the Co-Combustion Behavior of Semi-Coke and Typical Biomass: Combustion, NO Emission and Ash Characteristics Analysis. Fuel.

[38] Hashemi H., Hansen S., Toftegaard M.B., Pedersen K.H., Jensen A.D., Dam-Johansen K., Glarborg P. (2011). A Model for Nitrogen Chemistry in Oxy-Fuel Combustion of Pulverized Coal. Energy Fuels.

[39] Ma M., Liang Y., Xu D., Sun S., Zhao J., Wang S. (2022). Gas Emission Characteristics of Sewage Sludge Co-Combustion with Coal: Effect of Oxygen Atmosphere and Feedstock Mixing Ratio. Fuel.

[40] Gao C., Meng J., Liu Z., Hu Y., Wang J., Xu J., Wang H., Chen R. (2023). Effects of Water Hyacinth and Anthracite Co-Combustion under Different Conditions on SO_2_ and NOX Emission. Chem. Eng. Process.-Process Intensif..

[41] Wang X., Xu J., Ling P., An X., Han H., Chen Y., Jiang L., Wang Y., Su S., Hu S. (2023). A Study on the Release Characteristics and Formation Mechanism of SO_2_ during Co-Combustion of Sewage Sludge and Coal Slime. Fuel.

[42] Yan J., Wu Y., Zhang L., Huang S., Lei Z., Li Z., Zhang W., Ren S., Wang Z., Shui H. (2023). Synergistic Retention of Heavy Metals and In-Situ Reduction of NO and SO_2_ by Co-Combustion of Sewage Sludge and Coal Gangue: A Promising Approach for Contaminant Management and Emission Reduction. Fuel Process. Technol..

